# Retinal tauopathy as a biological indicator of Alzheimer’s disease

**DOI:** 10.4103/NRR.NRR-D-25-01096

**Published:** 2026-01-02

**Authors:** Bhakta Prasad Gaire, Yosef Koronyo, Keith L. Black, Dieu-Trang Fuchs, Maya Koronyo-Hamaoui

**Affiliations:** Department of Neurosurgery, Maxine Dunitz Neurosurgical Institute, Cedars-Sinai Medical Center, Los Angeles, CA, USA; Department of Biomedical Sciences, Division of Applied Cell Biology and Physiology, Cedars-Sinai Medical Center, Los Angeles, CA, USA; Department of Neurology, Cedars-Sinai Medical Center, Los Angeles, CA, USA

The retina, a laminated neural tissue at the back of the eye, is increasingly recognized as a site of central nervous system pathology in Alzheimer’s disease (AD) (Gaire et al., 2024). Developmentally derived from the embryonic diencephalon, the retina maintains a direct anatomical and physiological connection to the brain, comprising neurons, glial populations, and vascular structures that closely resemble those in the cerebral cortex. Both retina and brain originate from the neural ectoderm during embryogenesis and exhibit a layered cytoarchitecture composed of specialized neurons, supportive glia (astrocytes and microglia), and tightly regulated vasculature. These structures are protected by analogous blood-tissue barriers, the blood-retinal and blood–brain barriers, which mediate selective permeability and immune responses. While the brain is enclosed by the opaque skull and requires sophisticated, often expensive neuroimaging modalities for visualization, the retina is uniquely accessible through the transparent ocular media. This enables direct, high-resolution, noninvasive imaging of neuronal, glial, vascular, and molecular features *in vivo* using relatively affordable and clinically available technologies, including optical coherence tomography, scanning laser ophthalmoscopy, and increasingly, adaptive optics, hyperspectral imaging, and machine learning-based analytic platforms.

The unique convergence of shared neurobiology and anatomical accessibility with the brain has positioned the retina as a powerful and practical platform to interrogate pathology in the central nervous system. Given its homology with the brain and susceptibility to similar pathogenic mechanisms, including protein aggregation, synaptic loss, vascular compromise, oxidative stress, and chronic inflammation, the retina provides a unique window into neurodegenerative processes. In AD, emerging evidence from histological analyses, molecular assays, and advanced imaging has established that the cardinal pathological hallmarks — accumulation of amyloid β-protein (Aβ) deposits and inclusions of misfolded, hyperphosphorylated tau (p-tau) — are detectable in the AD retina (Gaire et al., 2024). Beyond pathogenic Aβ and tau forms, retinal vascular dysregulation, inflammatory responses, and neuronal degeneration have also been observed in patients with mild cognitive impairment (MCI) and AD dementia (reviewed in Gaire et al., 2024). These findings underscore the potential of the retina to serve not only as a biomarker site for early AD diagnosis but also as a surrogate for longitudinal monitoring and therapeutic intervention in a noninvasive, scalable, and repeatable manner. In particular, recent research has identified tauopathy as a robust and possibly early retinal correlate of cerebral disease (den Haan et al., 2018; Hart de Ruyter et al., 2023; Shi et al., 2024; Walkiewicz et al., 2024; Davis et al., 2025). In this perspective, we highlight the mounting evidence of retinal tauopathy in AD, including from our own recent studies (Shi et al., 2024; Davis et al., 2025), strengthening its potential as a clinically relevant biomarker for early AD detection and monitoring.

Among the hallmark lesions of AD, tau pathology has garnered increasing attention due to its strong correlation with neuronal dysfunction, cognitive impairment, and disease progression. Tau is a microtubule-associated protein encoded by the *MAPT* gene, which, in its physiological state, stabilizes microtubules and supports axonal transport. In the AD brain, tau undergoes a series of post-translational modifications, including hyperphosphorylation, truncation, citrullination, and conformational changes, which promote its dissociation from microtubules and self-assembly into toxic oligomers, paired helical filaments (PHFs), and eventually mature neurofibrillary tangles (NFTs). These aggregates disrupt cytoskeletal integrity, impair intracellular trafficking and neurotransmission, and lead to neuronal dysfunction and death.

A growing number of studies suggest that similar tau-related processes occur in the human retina (Koronyo et al., 2017; den Haan et al., 2018; Du et al., 2022; Hart de Ruyter et al., 2023; Shi et al., 2024; Walkiewicz et al., 2024; Davis et al., 2025). Histological analyses of postmortem retinal tissue from AD patients have consistently revealed the presence of total and p-tau species across multiple retinal layers. In addition to general tau detection using antibodies such as HT7 and 43D, studies have reported isoform-specific labeling of 3-repeat and 4-repeat tau, as well as a diverse panel of phosphorylated tau epitopes including AT8 (Ser202/Thr205), AT270 (Thr181), AT100 (Thr212/Ser214), pT217, pT231, and pS396 (den Haan et al., 2018; Du et al., 2022; Hart de Ruyter et al., 2023; Shi et al., 2024; Walkiewicz et al., 2024; Davis et al., 2025). These tau species exhibit specific spatial patterns within the retina, with prominent localization to the synapse-rich inner plexiform layer and outer plexiform layer (OPL) at the superior-temporal peripheral regions. Retinal p-tau species are also detected throughout the inner retinal layers, with considerable accumulation in the retinal nerve fiber layer, inner nuclear layer (INL), and ganglion cell layer (GCL). The distribution of these pathological tau isoforms in the retina mirrors early vulnerability regions in the brain, such as the entorhinal cortex and hippocampus, which are affected during the initial stages of AD.

Recent quantitative histopathological studies have further refined our understanding of retinal tau pathology. Hart de Ruyter et al. (2023) demonstrated that p-tau epitopes Ser202/Thr205 and Thr217 were markedly increased in INL neurons, specifically amacrine and horizontal cells, of AD patients compared to cognitively normal controls and non-AD neurodegenerative cases (Braak stage 0) (Hart de Ruyter et al., 2023). The burden of these tau species in the retina strongly correlated with Braak stage and the presence of NFTs in the hippocampus and cortex, indicating that retinal tauopathy may reflect parallel processes occurring in the brain. Other epitopes, such as Thr212/Ser214, Thr181, and Ser396, although detected, did not consistently differentiate AD from controls, highlighting the importance of selecting diagnostically informative isoforms for retinal biomarker development. A subsequent study further described the presence of these p-tau forms and additional abnormal tau species in the AD retina, including pTau231, Ser396/Ser404, 3R/4R tau, and MC-1-positive mature tau tangles, revealing that retinal primary tauopathy is associated with brain Aβ phase (Walkiewicz et al., 2024), suggesting a link between retinal tauopathy and cerebral amyloidosis in AD patients.

**Figure 1:**
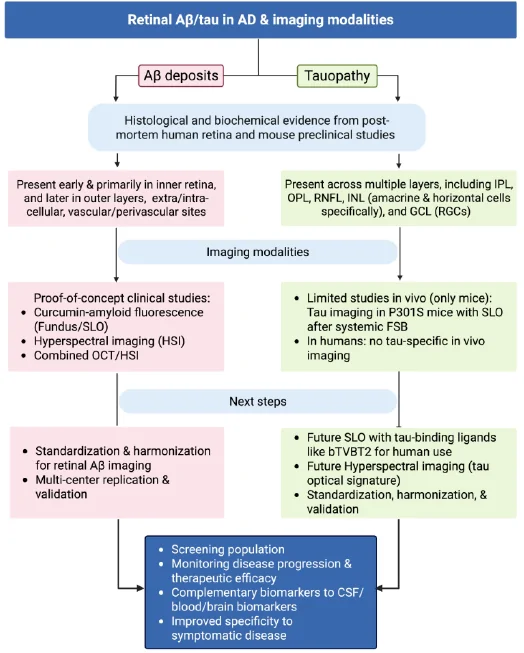
Retinal Aβ deposits and tauopathy in AD and imaging modalities. Schematic flowchart comparing evidence of pathogenic Aβ deposits and tau species, their retinal topography in AD, and a summary of existing and in-development imaging modalities. Created with BioRender.com. Aβ: Amyloid β-protein; AD: Alzheimer’s disease; bTVBT2: bi-thiophene-vinyl-benzothiazole; CSF: cerebrospinal fluid; FSB: trans,trans-1-fluoro-2,5-bis(3-hydroxycarbonyl-4-hydroxy)styrylbenzene; GCL: ganglion cell layer; HSI: hyperspectral imaging; INL: inner nuclear layer; IPL: inner plexiform layer; OCT: optical coherence tomography; OPL: outer plexiform layer; P301S: PS19 transgenic mice expressing the P301S mutant form of human microtubule-associated protein tau; PET: positron emission tomography; RGCs: retinal ganglionic cells; RNFL: retinal nerve fiber layer; SLO: scanning laser ophthalmoscopy.

Our recent study expanded these findings by quantitatively mapping the distribution of a broad array of tau isoforms in postmortem retinal tissues from patients with MCI (due to AD) and AD dementia, using immunohistochemistry and NanoString GeoMx digital spatial profiling (Shi et al., 2024). This multimodal approach enabled both spatial resolution and quantification of tau pathology. We assessed total tau, p-tau isoforms, citrullinated tau, T22-positive oligomeric tau (oligo-tau), and mature tau filaments and tangles (PHF-tau and MC-1). Our findings revealed a marked increase in pathological tau species in the retinas of both MCI and AD patients compared to individuals with normal cognition. Among these, retinal oligo-tau exhibited the highest fold-change: a 5.2-fold increase in MCI and a 9.2-fold increase in AD patients, and was 2.9-fold lower in non-AD dementia versus AD patients. Alongside elevated AT8-positive p-tau, CitR209-tau and pS396-tau were also significantly increased in MCI (3.5- and 2.2-fold, respectively) and AD (4.1- and 2.6-fold, respectively) retinas, with similar levels as non-AD dementia retinas. Mature tau conformers labeled by PHF-1 and MC-1 were less prevalent in the human retina, showing no-to-mild and 1.8–2.3-fold increases in MCI and AD retinas, respectively, suggesting a temporal progression from pretangle to mature forms in the dementia phase. Notably, GeoMx profiling in another human cohort revealed increases in retinal p-tau at sites S214, S396, S404, and T231. These retinal p-tau forms were disproportionately elevated in MCI compared to AD, suggesting that retinal tauopathy accumulates early in the disease course and may serve as biomarkers for prodromal AD. Importantly, levels of retinal oligo-tau and pS396 significantly correlated with clinical and neuropathological indices, such as brain NFT severity, Braak stage, ABC score, and cognitive dysfunction assessed by clinical dementia rating or mini-mental state examination (Shi et al., 2024). Concomitant with these results, a recent study utilizing mass-spectrometry in postmortem human retina and hippocampus showed elevated retinal p-tau (e.g., S199/S202, T231, and S396^+^T403/S404) in AD, with site-specific correlations to brain Aβ/NFT, Braak stages, and to hippocampal p-tau, notably S396^+^T403/S404 (Santiago et al., 2025). Together, these findings show that retinal tauopathy, along with Aβ signatures, track cerebral pathology and overall disease status, supporting their utility as biomarkers for AD diagnosis and monitoring (**Figure 1**).

Across the brain, Aβ deposition has been shown to trigger and accelerate tauopathy, promoting phosphorylation, oligomerization, and trans-neuronal spread, indicating that these pathologies act synergistically rather than independently. In the AD retina, we observed a similar coupling: oligo-tau correlates very strongly with Aβ_42_ and arterial Aβ_40_, CitR209-tau tracks tightly with Aβ_42_, and AT8^+^ p-tau shows moderate associations with Aβ_42_/Aβ_40_ and Aβ oligomers. Together with histological hints of early tauopathy in synaptic/axonal-rich layers (OPL to inner plexiform layer) with further spread to INL and GCL/NFL, and peripheral to central trajectories within the superior-temporal retina, these findings support an Aβ-driven tau propagation model that warrants future studies, including quantitative, layer- and quadrant-resolved analyses. Notably, distinct distribution patterns of Aβ and tau deposition were observed in retinas of MCI and AD patients. Aβ_42_ appears earliest and most densely in the inner retinal layers, predominantly across the mid- and far-periphery of the superior and inferior-temporal retina, extending toward outer layers in AD dementia stages. In contrast, tau exhibits a staged spatiotemporal distribution: in MCI, upregulated pS396-tau and abundant oligo-tau span from OPL into inner layers, with CitR209-tau already increased and co-localizing with AT8^+^ pS202/T205-tau in OPL/INL; by AD dementia, conformational PHF-tau, and occasionally MC-1^+^ NFT-like “paperclip” tau tangles, become more prominent within neurons of INL/GCL and OPL and extend across inner plexiform layer and ONL. These Aβ and tau deposition patterns, mirrored by layer thickness loss in the same peripheral retinal regions, support the clinical utility of early tau species (oligomeric, citrullinated, and hyperphosphorylated) in the same regions that first harbor Aβ_42_, thereby sharpening stage-specific retinal diagnostics beyond Aβ mapping alone.

In a later study, we investigated the involvement of retinal ganglion cells (RGCs) in tau-related pathology and neurodegeneration (Davis et al., 2025). Using the RGC-specific marker RNA-binding protein with multiple splicing, we identified a 42%–65% reduction in RGC counts in MCI and AD retinas compared to controls, with the most pronounced degeneration observed in mid-peripheral subregions of the superior-temporal retina. These findings were corroborated by Nissl staining, which revealed 55%–56% total neuronal loss in the GCL, particularly in these mid-peripheral zones. Morphological abnormalities observed in surviving RNA-binding protein with multiple splicing-positive RGCs included soma hypertrophy, nuclear displacement, and evidence of charged multivesicular body protein 2B-positive granulovacuolar degeneration bodies, a hallmark of stressed neurons. Further, we detected granulovacuolar degeneration-associated necroptotic marker by phosphorylated mixed lineage kinase domain-like protein labeling, indicating that these RGCs were undergoing regulated cell death pathways in response to chronic AD-related injury.

Moreover, a substantial proportion of RGCs in MCI and AD retinas were positive for cleaved caspase-3, an early apoptotic marker. Quantitative analysis revealed a 1.3–1.8-fold increase in cleaved caspase-3-positive RGCs, with apoptotic RGCs comprising 25%–30% of the total RGC population in diseased retinas. Critically, pathological tau isoforms, particularly oligo-tau and pS396-tau, were localized within RGCs and were significantly elevated in MCI and AD compared to normal cognition controls. In fact, pS396- or oligo-tau-laden RGCs were 2.4–3.5-fold more abundant in these patients. PHF-1-positive tau was less frequently detected in RGCs, indicating a predominance of pretangle forms in these neurons. Notably, tauopathy-positive RGC counts correlated inversely with RGC density and directly with retinal cleaved caspase-3 as well as cerebral Aβ-plaque, NFT, Braak, and mini-mental state examination scores. In advanced-stage AD patients (Braak V–VI, clinical dementia rating = 3), tau-positive RGCs were more prevalent, particularly in mid-peripheral retina, where oligo-tau burden exhibited the strongest association with cognitive performance and neuropathological severity (Davis et al., 2025). Overall findings establish RGCs as early targets of retinal tauopathy in AD and suggest that pretangle tau forms, rather than mature tangles, may mediate cytotoxicity via both apoptotic and necroptotic pathways. Hence, tauopathy-laden RGCs may serve as cellular indicators of early and progressive neurodegenerative changes in AD. Yet, despite strong associations in human retina and supportive preclinical data, causality between tau pathology and RGC degeneration in AD remains to be established.

Additional studies have implicated retinal glial cells in tauopathy. Nunez-Diaz et al. (2024) demonstrated that retinal microglia internalize p-tau (AT8 and PHF-1) in AD retinas, suggesting potential roles in tau clearance, propagation, or inflammation. Furthermore, advances in retinal imaging technologies are beginning to capture these pathological changes *in situ*. Using hyperspectral imaging, we detected unique spectral signatures of pS396-tau in unstained postmortem retinal tissues, demonstrating a strong correlation between optical signals and immunolabeled pathology (Du et al., 2022). Notably, deep learning-based analysis pipelines could predict the presence of p-tau in retinal sections, supporting the feasibility of noninvasive tau imaging as a future diagnostic modality (Du et al., 2022). In preclinical AD models, pathological tau species accumulate early within the retinal GCL and ONL, preceding cognitive decline and cerebral tauopathy (Gaire et al., 2024), with evidence suggesting prion-like propagation and temporal interplay with Aβ deposition, underscoring its biomarker potential.

Despite the accumulating evidence, certain limitations remain. To date, only two studies have visualized NFT-like structures in the human retina using silver-based histochemical staining and MC-1-positive immunolabeling (Koronyo et al., 2017; Shi et al., 2024). Although these studies documented a broad range of tau species, the precise ultrastructural characterization of retinal NFTs remains unexplored. Moreover, the mechanisms underlying tau propagation within retinal neurons and across synaptic layers remain poorly understood. The relationships between retinal tauopathy and concurrent retinal processes, such as Aβ accumulation, vascular amyloidosis, gliosis, and oxidative stress, require further investigation to determine whether these phenomena act synergistically or independently in the pathogenesis of retinal neurodegeneration. Clinically, future studies should assess whether retinal tauopathy correlates with brain and fluid biomarkers in AD.

Taken together, the evidence of shared pathophysiological vulnerabilities positions retinal tauopathy as a biologically and clinically relevant extension of AD pathology. As research continues to refine our understanding of retinal tau species, including their cellular localization and temporal progression, the potential to develop noninvasive, retinal-based diagnostics for early detection, risk stratification, and treatment monitoring in AD becomes increasingly tangible. The integration of spatial transcriptomics, multiplex immunohistochemistry, and machine learning-assisted imaging may soon enable precise, personalized assessment of tauopathy through a simple retinal scan, ushering in a new era of neurodegenerative disease diagnostics.

*This article is dedicated to the memory of Dr. Salomon Moni Hamaoui and Lillian Jones Black, both of whom passed away from Alzheimer’s disease.*

*This work was supported, in part, by the National Institutes of Health (NIH)/National Institute on Aging (NIA) grant numbers: R01AG056478, R01AG055865, and R01AG075998 (to MKH). Additionally, the work was supported by the Snyder, Jona Goldrich, Gordon Foundations, and the Hertz Innovation Fund (to MKH)* *.*
